# The Antiproliferative Activity of Oxypeucedanin via Induction of G_2_/M Phase Cell Cycle Arrest and p53-Dependent MDM2/p21 Expression in Human Hepatoma Cells

**DOI:** 10.3390/molecules25030501

**Published:** 2020-01-23

**Authors:** So Hyun Park, Ji-Young Hong, Hyen Joo Park, Sang Kook Lee

**Affiliations:** College of Pharmacy, Natural Products Research Institute, Seoul National University, Seoul 08826, Korea; hanirela@snu.ac.kr (S.H.P.); jyhong7876@snu.ac.kr (J.-Y.H.); phj00@snu.ac.kr (H.J.P.)

**Keywords:** oxypeucedanin, *Angelica dahurica*, antiproliferation, G_2_/M phase cell cycle arrest, p53, SK-Hep-1, hepatoma cells

## Abstract

Oxypeucedanin (OPD), a furocoumarin compound from *Angelica dahurica* (Umbelliferae), exhibits potential antiproliferative activities in human cancer cells. However, the underlying molecular mechanisms of OPD as an anticancer agent in human hepatocellular cancer cells have not been fully elucidated. Therefore, the present study investigated the antiproliferative effect of OPD in SK-Hep-1 human hepatoma cells. OPD effectively inhibited the growth of SK-Hep-1 cells. Flow cytometric analysis revealed that OPD was able to induce G_2_/M phase cell cycle arrest in cells. The G_2_/M phase cell cycle arrest by OPD was associated with the downregulation of the checkpoint proteins cyclin B1, cyclin E, cdc2, and cdc25c, and the up-regulation of p-chk1 (Ser345) expression. The growth-inhibitory activity of OPD against hepatoma cells was found to be p53-dependent. The p53-expressing cells (SK-Hep-1 and HepG2) were sensitive, but p53-null cells (Hep3B) were insensitive to the antiproliferative activity of OPD. OPD also activated the expression of p53, and thus leading to the induction of MDM2 and p21, which indicates that the antiproliferative activity of OPD is in part correlated with the modulation of p53 in cancer cells. In addition, the combination of OPD with gemcitabine showed synergistic growth-inhibitory activity in SK-Hep-1 cells. These findings suggest that the anti-proliferative activity of OPD may be highly associated with the induction of G_2_/M phase cell cycle arrest and upregulation of the p53/MDM2/p21 axis in SK-HEP-1 hepatoma cells.

## 1. Introduction

Hepatocellular carcinoma (HCC) is the sixth most frequently diagnosed cancer and the second most common cause of death from cancer [1]. Approximately 30 to 40% of patients with HCC diagnosed at early stages are potentially effectively treated by surgical therapies such as hepatectomy or liver transplantation [2]. However, the disease diagnosed at an advanced stage or re-progression stage after treatment has poor prognosis due to the fundamental liver disease and lack of other treatment options [2,3,4]. In accordance, any systemic therapy has not effectively contributed to survival for patients with advanced HCC [5]. Therefore, it is necessary to procure potential chemotherapeutic agents for the treatment of advanced HCC cells.

**Figure 1:**
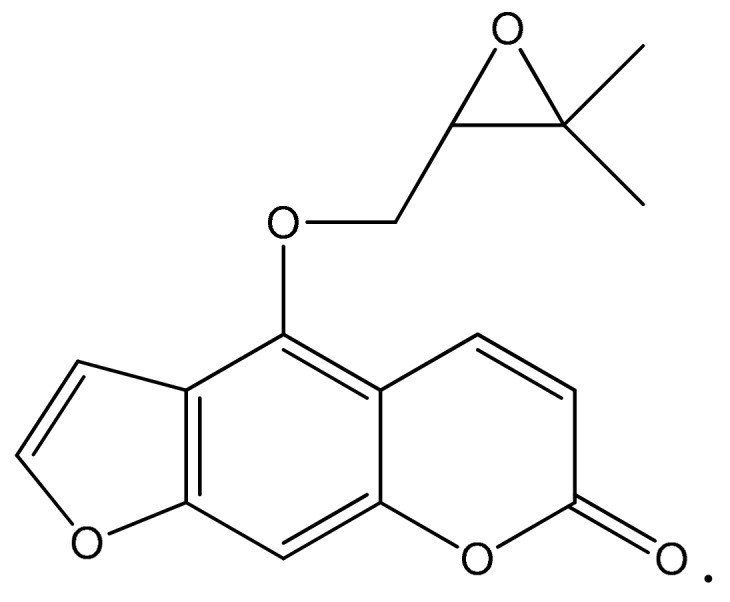
Chemical structure of oxypeucedanin (OPD).

*Angelica dahurica* (Umbelliferae) is an indigenous plant mainly distributed in Korea, China, and Russia. The root of *Angelica dahurica* has been used for the control of hysteria, bleeding, menstrual disorder, neuralgia and pain as a traditional medicine in Korea. Previous phytochemical studies revealed that the plant is a rich source of furanocoumarins, including oxypeucedanin [6]. Oxypeucedanin (OPD) (Figure 1), a coumarin-type major constituent of the root of *Angelica dahurica*, has been reported to have several biological activities, including anti-mutagenic, uterus contraction, blood pressure increase, and cytotoxic and antiproliferative effects against cancer cells [7,8,9].

When the DNA damage stimulus comes to the cells, the response of the cellular system is complex and finely controlled. The cellular response involves the functions of gene products that recognize DNA damage signals and activate processes such as the inhibition of proliferation, the stimulation of repair mechanisms, or the induction of apoptosis [10,11,12]. Generally, the cellular response to DNA damage and the disturbance to replication involve the activation of checkpoints, following signal transduction pathways for regulation of cell cycle progression and cell division [10,11]. Deficiency in these checkpoint responses can induce cell death, genomic instability, or predisposition to cancer [12,13,14]. In addition, cell cycle progression is finely regulated by cell cycle regulatory proteins and checkpoint proteins depending on the specific cell cycle phase. In particular, the G_2_/M phase of the cell cycle is governed by the expression of key G_2_/M transition regulatory proteins and the ATR/Chk1 signaling pathways. p53, a tumor suppressor, is also considered to be involved in the transcriptional regulation of a large number of growth-arrest and apoptosis-related genes [15].

Although several biological activities of OPD have been previously reported, the precise molecular mechanism of OPD related to its antiproliferative activity against human liver cancer cells has not been fully elucidated. In the present study, the growth-inhibitory activity and the underlying mechanisms of action of OPD were investigated in SK-Hep-1 human hepatoma cells.

## 2. Results

### 2.1. Antiproliferative Effects of Oxypeucedanin (OPD) in SK-Hep-1 Human Hepatoma Cells

To primarily evaluate whether OPD shows potential growth-inhibitory effects on human cancer cells, the anti-proliferative activity of OPD was determined in a panel of human cancer cell lines. As summarized in Table 1, OPD inhibited the growth of human cancer cells. Among the cell lines tested, SK-Hep-1 human hepatoma cells were the most sensitive to OPD. Therefore, further study on the mechanism of action of OPD in the regulation of cell proliferation was conducted in SK-Hep-1 cells. In addition, since OPD was shown to have the anti-proliferative activity against SK-Hep-1 cells, the isolated OPD analogs from the roots of *Angelica dahurica* were also evaluated for their antiproliferative activity in SK-Hep-1 cells. Among the test compounds, OPD was the most active growth inhibitor against SK-Hep-1 cells (Table 2).

**Figure 2:**
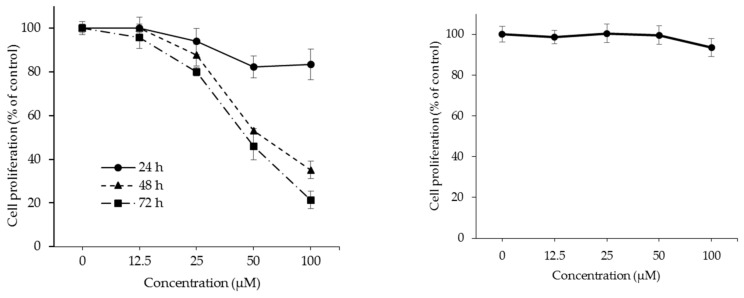
Anti-proliferative effects of OPD in SK-Hep-1 human hepatoma cells (left) and MRC5 human normal cell line (right). SK-Hep-1 cells were cultured in a 96-well plate and treated with the indicated concentrations of OPD for 24–72 h. MRC5 cells were cultured in a 96-well plate and treated with the indicated concentrations of OPD for 72 h. The cell proliferative activity was determined using the SRB assay. The % cell proliferation value was calculated by the mean absorbance of samples/absorbance of the vehicle-treated control as described in the Materials and Methods. Data are presented as the means ± S.E. (*n* = 3).

Since the anti-proliferative activity of OPD was found in SK-Hep-1 human hepatoma cells for 72 h, the growth-inhibitory activity of OPD was also investigated for 24 or 48 h in SK-Hep-1 cells. As shown in Figure 2, OPD exhibited growth-inhibitory activity against SK-Hep-1 human hepatoma cells in a concentration- and time-dependent manner.

The IC_50_ value of OPD with a 72 h treatment was 32.4 μM. In addition, the growth-inhibitory activity of OPD was also determined in a normal cell line. OPD was unable to affect the growth rate of MRC5 normal human lung fibroblast cells (IC_50_ >100 µM). These data suggest that OPD may be able to selectively inhibit the proliferation of human hepatoma cancer cells compared to normal cells. Under the same experimental conditions, the IC_50_ value of etoposide, a positive control, was 0.3 μM.

### 2.2. Effects of OPD on the Cell Cycle Distribution of SK-Hep-1 Cells

**Figure 3:**
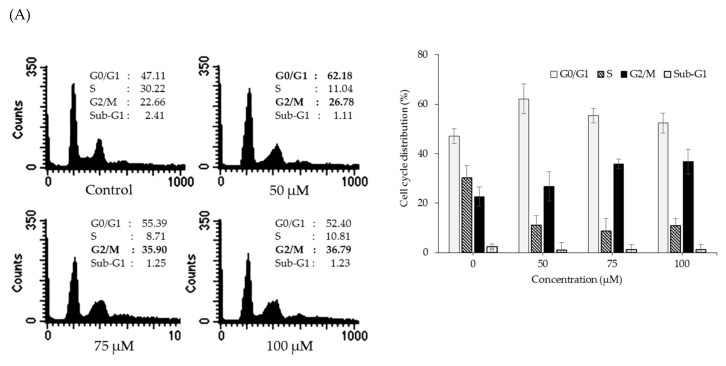

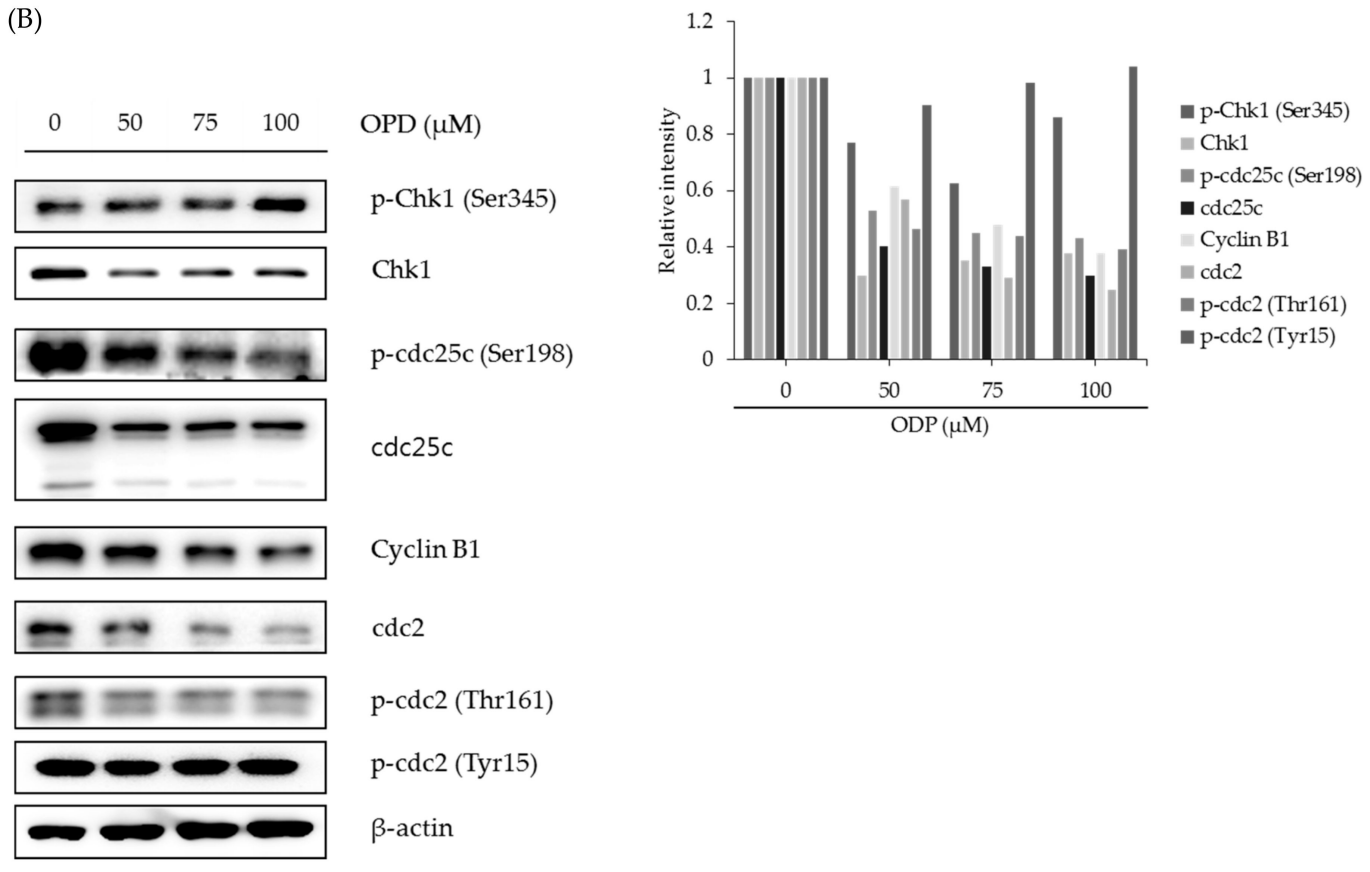
Effects of OPD on the regulation of cell cycle distribution in SK-Hep-1 cells. (**A**) SK-Hep-1 cells were treated with various concentrations of OPD for 24 h. Both adherent and floating cells were collected, fixed with 70% cold ethanol overnight, and then incubated with RNase A and PI for 30 min. The cell cycle distribution was analyzed by flow cytometry. (**B**) The cells were treated with the indicated concentrations of OPD for 24 h, and the expression of cell cycle regulatory proteins was determined by western blot analysis. β-Actin was used as an internal control.

To further elucidate the anti-proliferative mechanisms of OPD in SK-Hep-1 cells, the cells were treated with the indicated concentrations of OPD for 24 h, and flow cytometry analysis was performed with PI staining. As shown in Figure 3A, OPD enhanced the accumulation of the G_2_/M phase peak from 22.66% (control) to 35.90% (75 μM). These data suggest that the antiproliferative activity of OPD in SK-Hep-1 cells is in part associated with the induction of G_2_/M phase cell cycle arrest. To further investigate whether the G_2_/M phase cell cycle arrest by OPD is correlated with the regulation of the checkpoint proteins, the expression of the G_2_/M cell cycle regulatory proteins was determined by western blot analysis. Since OPD did not show significant cytotoxicity at the test concentration up to 100 μM for 24 h (Figure 2), the cells were treated with OPD (50, 75, or 100 μM) for 24 h, and then the checkpoint protein expression related to G_2_/M phase cell cycle regulation was measured in SK-Hep-1 cells. As shown in Figure 3B, the expression levels of Chk1, p-cdc25c (Ser198), cdc25c, cyclin B1, cdc2, and p-cdc2 (Thr161) were downregulated, but the levels of p-Chk1 (Ser345) were upregulated by OPD treatment. Chk1 (checkpoint kinase 1) is a multifunctional protein kinase that coordinates the response to specific types of DNA damage [16]. Cdc25 is a protein phosphatase responsible for dephosphorylating and activating cdc2, a pivotal step in directing the cells toward mitosis [17]. When DNA damage ocurrs, the Chk1 phosphorylates cdc25c, which then leads to translocation of cdc25c from the cytoplasm to the nucleus, where cdc25c can interact with cdc2/cyclin B during mitosis [18,19]. Moreover, the activity of the cdc2-cyclin B1 complex is dependent on the phosphorylation/dephosphorylation status of cdc2 [11,13,20]. The entry of eukaryotic cells into mitosis is regulated by cdc2 activation, including the binding of cdc2 to cyclin B1 and its phosphorylation at the Thr161 residue. In this study, we found that cdc25c was inactivated by phosphor-Chk1 with OPD treatment, and the activation of the cdc2-cyclin B1 complex was also suppressed by OPD in a concentration-dependent manner, indicating the induction of G_2_/M phase cell cycle arrest by OPD. These findings suggest that the activation of Chk1 and sequential regulation of signal transduction pathways by OPD may be due to the induction of G_2_/M phase cell cycle arrest by OPD in SK-Hep-1 cells.

### 2.3. Effects of OPD on the Regulation of p53-associated Signaling Pathways in SK-Hep-1 Cells

**Figure 4:**
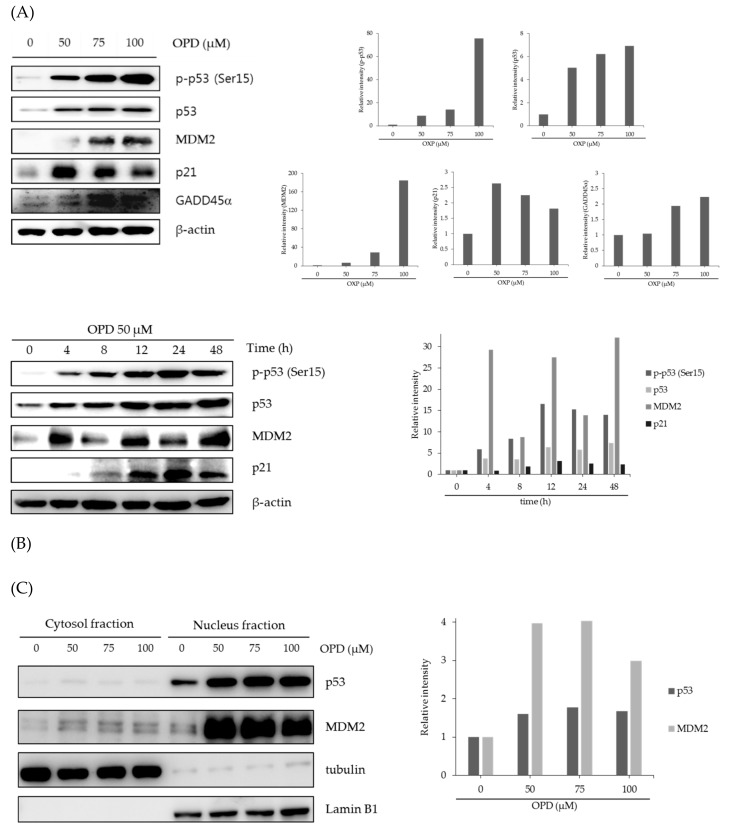

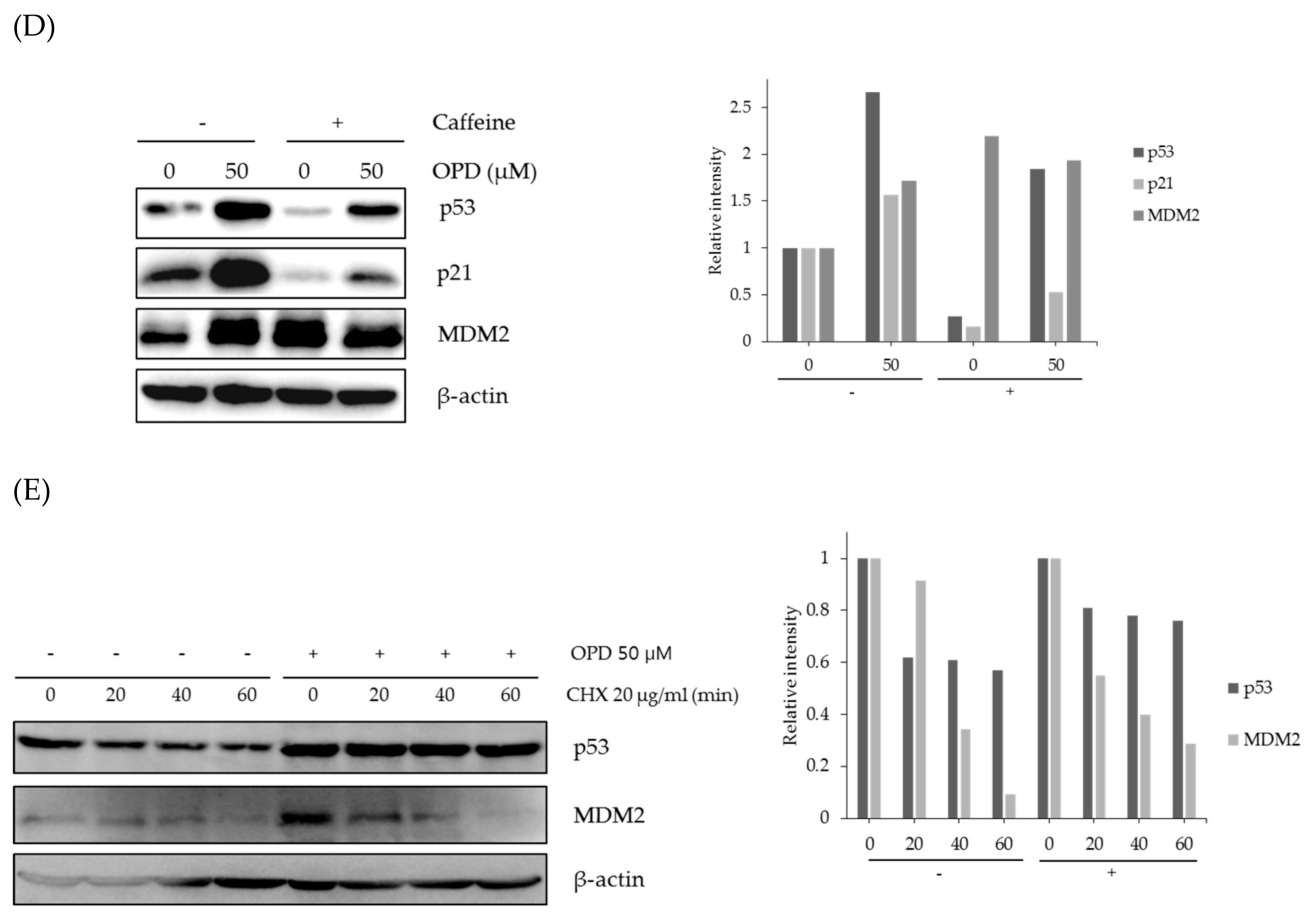
Effects of OPD on the regulation of p53-associated signaling pathways. (**A**) SK-Hep-1 cells were treated with the indicated concentrations of OPD for 24 h. The expression level of proteins was analyzed by western blot. β-Actin was used as an internal control. (**B**) SK-Hep-1 cells were treated with 50 μM OPD for various time points up to 48 h. The expression level of proteins was analyzed by western blot. β-Actin was used as an internal control. (**C**) SK-Hep-1 cells were treated with various concentrations of OPD for 24 h. After cells were harvested, the proteins were separated into cytosolic and nuclear fractions as described in the Materials and Methods. The protein expression levels were analyzed by western blot. β-Actin was used as an internal control. (**D**) SK-Hep-1 cells were pretreated for 2 h with 1 mM caffeine, and then cultured with 50 μM OPD for an additional 24 h. The expression level of proteins was analyzed by western blot. β-Actin was used as an internal control. (**E**) SK-Hep-1 cells were treated with 50 μM OPD for 24 h and then treated in the absence or presence of 20 μg/mL cycloheximide. The cells were harvested at 0, 20, 40, and 60 min. The expression level of proteins was analyzed by western blot, and the band density was quantified using the NIH ImageJ software (Bethesda, MD).

To further validate the association of p53-mediated signaling molecules in the anti-proliferative activity of OPD in SK-Hep-1 cells, the cells was treated with OPD for 24 h, and then the expression of p53-associated molecules were determined by western blot analysis. OPD significantly enhanced the protein expression of p53, p-p53(Ser15), MDM2, p21, and GADD45α in the cells (Figure 4A). Previous studies have reported that the phosphorylation of p53 at Ser15 or Ser20 upregulates p53 stability by disrupting the interaction between p53 and MDM2 [21,22]. Because OPD is able to induce the stability of p53, the accumulation of p53 by OPD may subsequently enhance its downstream target genes, including MDM2, p21, and GADD45α, in the cells. In addition, the expression of p53-associated proteins was also monitored for up to 48 h with treatment with 50 μM OPD and was found to be up-regulated after 4 h of treatment in SK-Hep-1 cells (Figure 4B). Further study was designed to confirm whether the cellular localization of p53 is affected by treatment of cells with OPD. Generally, in the nucleus, MDM2-mediated ubiquitination led to the transportation of p53 into the cytoplasm or its degradation by the 26S proteasome [21,22]. As an important transcriptional factor, the stability and nuclear localization of p53 are considered essential for its tumor suppressor activity [23]. Therefore, we determined the levels of p53 and MDM2 protein expression in both the cytosol and nucleus fraction following treatment with OPD in SK-Hep-1 cells. As shown in Figure 4C, the levels of p53 protein expression were upregulated and localized in the nucleus upon OPD treatment for 24 h in a concentration-dependent manner. The levels of MDM2 protein expression were also shown to be upregulated in the nucleus by OPD treatment in the cells. To further confirm the effects of OPD on the expression of p53-associated proteins, OPD treatment of cells was conducted in the absence or presence of caffeine. Because caffeine is known as an ATR/Chk1 kinase inhibitor [24,25], the effect of caffeine on the enhanced p53-associated protein expression by OPD was investigated in the cells. As expected, treatment with OPD (50 μM) for 24 h led to a significant upregulation of p53, p21, and MDM2 expression in the absence of caffeine, but the pretreatment with caffeine for 2 h alleviated the enhancement of p53-associated protein expression by OPD in SK-Hep-1 cells (Figure 4D). Although the expression of p53-associated proteins was downregulated by the pretreatment with caffeine, OPD also exhibited a slight upregulation of p53 and p21 expression in the presence of caffeine (Figure 4D). These data suggest that the induction of G_2_/M phase cell cycle arrest by OPD seems to be associated with not only the ATR/Chk1 signaling pathway but also the regulation of p53-mediated pathways. Further study examined the maintenance of p53 stability by OPD with the cotreatment of cycloheximide (CHX), a protein synthesis inhibitor [26], in SK-Hep-1 cells. As shown in Figure 4E, OPD significantly suppressed the degradation of p53 in the presence of cycloheximide, suggesting that the half-life of p53 degradation was sustained by OPD treatment compared to vehicle-treated control cells. However, the rate of MDM2 degradation was not greatly affected by the down-regulation of p53 turnover with OPD treatment of cells.

### 2.4. Effects of OPD on Cell Proliferation Depending on the p53 Status in Hepatoma Cells

**Figure 5:**
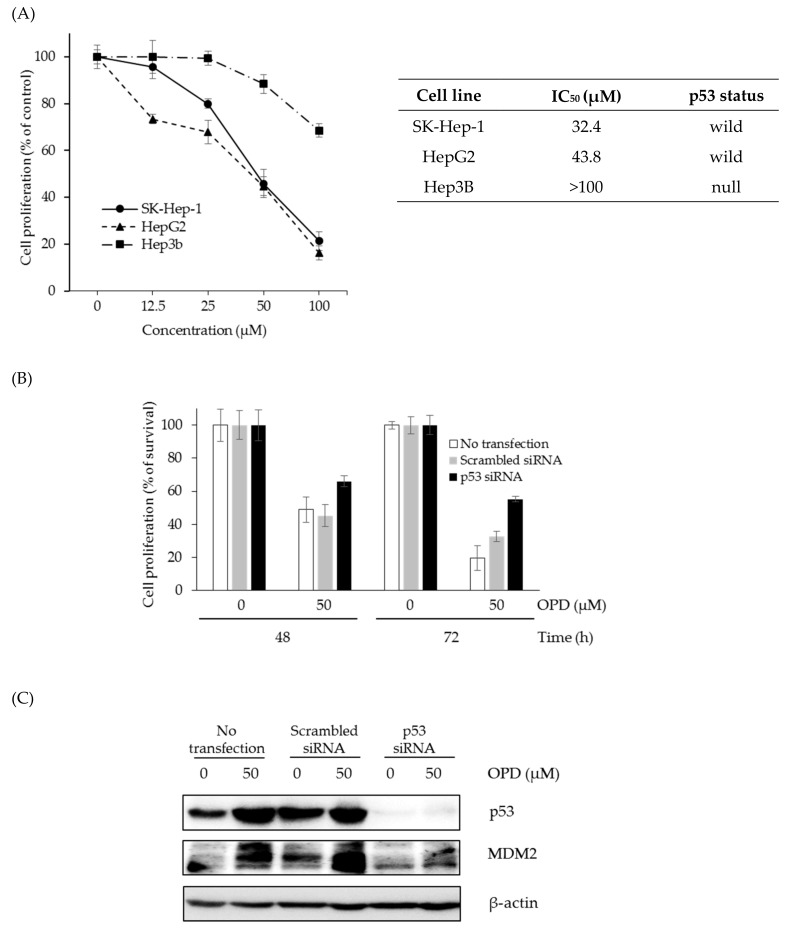
Effects of p53 expression on the cell proliferation of hepatoma cells by OPD. (**A**) SK-Hep-1, HepG2, and Hep3B cells were cultured in a 96-well plate and treated with the indicated concentrations of OPD for 72 h. The cell proliferative effect was determined using the SRB assay. Data are presented as the means ± S.E. (*n* = 3). (**B**) SK-Hep-1 cells were transfected with 5 nM p53 siRNA or scrambled siRNA for 24 h, and then OPD treatment (50 μM) was given for 48 or 72 h for the measurement of cell proliferation. Data are presented as the means ± S.E. (*n* = 3). (**C**) The effects of p53 siRNA on the expression level of p53 and MDM2 were analyzed by western blot in control cells and p53 siRNA-transfected SK-Hep-1 cells.

Since p53 seems to be highly associated with the anti-proliferative activity of OPD in SK-Hep-1 cells, the effects of p53 status on the growth-inhibitory activity of OPD were evaluated in human hepatoma cells with different p53 statuses. The cells were treated with various concentrations of OPD for 72 h, and then cell proliferation was determined by the SRB assay. As shown in Figure 5A, wild-type p53 cell lines, such as SK-Hep-1 and HepG2, were more sensitive than the p53-null Hep3B cell line, indicating that p53 plays an important role in the anti-proliferative activity of OPD in human hepatoma cells. To further confirm the involvement of p53 in the antiproliferative activity of OPD, p53 siRNA was transfected into wild-type p53 SK-Hep-1 cells, and the effect of p53 siRNA on the cell proliferation was determined in SK-Hep-1 cells. When the p53 siRNA transfected-SK-Hep-1 cells were treated with OPD (50 μM) for 48 and 72 h, the antiproliferative activity of OPD was decreased compared to that of the control cells (no transfection or scrambled siRNA transfection), suggesting that the anti-proliferative activity of OPD is partly associated with wild-type p53 expression in hepatoma cells (Figure 5B). In addition, the OPD treatment exhibited upregulation of p53 and MDM2 in the control cells. Although p53 expression was not shown in p53 siRNA-treated SK-Hep-1 cells, OPD slightly up-regulated the expression of MDM2 in p53 siRNA-treated SK-Hep-1 cells (Figure 5C).

### 2.5. Effects of OPD in Combination with Gemcitabine on the Cell Proliferation of SK-Hep-1 Cells

**Figure 6:**
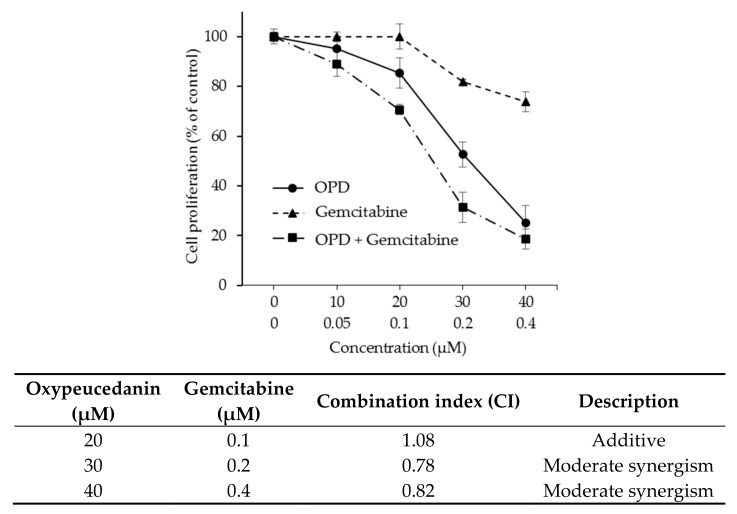
Combination effect of OPD with gemcitabine on the cell proliferation of SK-Hep-1 cells. (**A**) The cells were treated with the indicated concentrations of OPD and gemcitabine for 72 h, and then cell proliferation was measured by the SRB assay. (**B**) The cells were treated with OPD (20 μM) and various concentrations of gemcitabine (0.1–0.4 μM) for 72 h, and then cell proliferation was measured by the SRB assay. Data are presented as the means ± S.E. (*n* = 3). Based on the cell proliferation data.

To further investigate the effect of the combination of OPD with an anticancer agent on the antiproliferative activity of hepatoma cells, we selected gemcitabine, a pyrimidine-based DNA synthesis inhibitor, as a candidate compound based on the use of patients with liver cancer in the clinic. As shown in Figure 6A, cotreatment with OPD and gemcitabine exhibited a synergistic effect (CI < 1.0) on the antiproliferative activity of SK-Hep-1 cells. The combination of the higher concentrations of OPD and gemcitabine showed a relatively stronger synergistic activity. In accordance, when OPD (20 μM) was treated with various concentrations of gemcitabine (0.1–0.4 μM), the combination with the higher concentration of gemcitabine (0.4 μM) was shown to have a stronger synergistic activity (Figure 6).

Natural products have been used for traditional medicines and also play an important role in drug discovery and development programs. In particular, anticancer drugs are mainly developed based on natural product-originated small molecules. In our continuous efforts to procure small molecules from natural sources in the discovery of antitumor agents, oxypeucedanin (OPD), a furanocoumarin isolated from the root of *Angelica dahurica,* was considered a potential candidate. Previous biological activities of OPD include the effective intervention of sunitinib-induced nephrotoxicity, the hepatoprotective activity of tacrine-induced cytotoxicity in liver cells, and the cytotoxicity against cancer cells such as gastric cancer, prostate cancer, and melanoma cells [27,28]. Although the antiproliferative activities of OPD have been reported in cancer cells, the precise molecular mechanism of OPD in the anticancer activity of human liver cancers remains to be elucidated. This study demonstrates that OPD regulates the induction of G_2_/M phase cell cycle arrest and p53-dependent MDM2/p21 signaling pathways in human hepatoma SK-Hep-1 cells.

It is known that the cell cycle is a finely controlled sequence of events in the growth and proliferation of eukaryotic cells. Cell cycle progression occurs in an ordered manner that is monitored by cell cycle checkpoints. Among the checkpoints, the DNA damage-induced G_2_/M checkpoint guarantees the fidelity of genomic stability. However, cancer cells have abnormally activated in cell division as a result of diverse factors, including uncontrolled checkpoint protein expression. These defects in the checkpoints lead to genomic instability, cell death, or carcinogenesis. Therefore, the regulation of the G_2_/M checkpoint is often applied as a potential therapeutic target to evaluate the efficacy of natural product-derived antitumor agents in cancer cells. In the present study, we found that OPD effectively inhibited the growth of SK-Hep-1 human hepatoma cells. Cell cycle distribution analysis also revealed that the antiproliferative activity of OPD is in part associated with the induction of G_2_/M cell cycle arrest in cells. This result was consistent with the previous report of G_2_/M phase arrest of OPD in prostate cancer cells [27]. However, a previous study of the antiproliferative activity of OPD on the prostate cancer cells did not investigate in detail the regulatory biomarkers involved in G_2_/M phase cell cycle arrest in cancer cells. The present study revealed that the induction of G_2_/M phase cell cycle arrest by OPD was correlated with the regulation of Chk1-mediated G_2_/M checkpoint proteins, including cdc25c/cdc2 and cdc2/cyclin B1 complex pathways, in hepatoma cells. Chk1 is considered to play an important role in the G_2_/M checkpoint via the ATM-RAD3-related (ATR) pathway. Chk1 also regulates the activity of its shared downstream substrate, cell division cycle 25c (cdc25c). OPD effectively modulates the Chk1-cdc25c activation pathway axis, leading to the induction of G_2_/M cell cycle arrest in SK-Hep-1 cells. We also found that OPD effectively suppressed the activity of cyclin B1 and its partner cell division cycle 2 (cdc2) expression, which in turn evokes the prevention of entry into the mitosis (M) phase cell cycle transition in hepatoma cells.

Tumor suppressor p53 is mutated, deleted, or rearranged in more than half of all human tumors, and thus, p53 is considered an important target in anticancer drug development [29,30]. p53 is also considered an important factor in the control of the cell cycle at the G_1_/S and/or G_2_/M transition through diverse mechanisms [31]. In this study, we found that OPD significantly up-regulated the expression of p53 by the accumulation of p53 and increased its stability in SK-Hep-1 cells. The upregulation of p53 levels by OPD led to the activation of downstream target expression such as MDM2, p21 and GADD45α in cells. The association of p53 in G_2_/M phase cell cycle signaling was further confirmed by the significant suppression of p53, p21 and MDM2 expression by treatment with caffeine, an ATR/Chk1 inhibitor, in SK-Hep-1 cells. However, OPD did not fully recover the levels of p53 and its downstream target protein expression in the presence of caffeine, indicating that OPD may cause the activation of p53 independently of the ATR/Chk1 pathway. The involvement of p53 in the anti-proliferative activity of OPD in SK-Hep-1 cells was also confirmed using cell types with different p53 statuses and deletion methods. OPD was found to be more effective in p53-wild-type expression cells, and p53 siRNA-transfected cells were less sensitive to the OPD in the antiproliferative activity on liver cancer cells.

The function of p53 is mainly governed by the stability and nuclear localization of p53 in cells. We found that OPD effectively inhibits the degradation of p53 and thus sustains the stability of p53 in cancer cells. In addition, OPD was found to induce the nuclear localization of p53, which may activate the transcriptional activity of its downstream target genes associated with cell cycle regulation in cancer cells.

Gemcitabine (2′,2′-difluorodeoxycytidine), a pyrimidine-based antimetabolite, is currently used to the treatment of pancreatic cancer as well as other cancers [32,33]. Gemcitabine inhibits DNA replication by activating the S-phase checkpoint. A study also showed that gemcitabine activates the ATR/Chk1 pathway [34]. In the present study, we found that OPD in combination with gemcitabine exhibits synergistic anti-proliferative activity in SK-Hep-1 cells. Therefore, OPD might be a promising lead compound in combination with cancer chemotherapeutic agents in advanced liver cancer treatment. In addition, in terms of clinical relevance of furanocoumarins, the bioavailability of oxypeucedanin in plasma samples may be important parameters. In the previous study, Chen et al. [35] reported that the pharmacokinetic parameters of OPD were the Tmax (12 h), T1/2 (2.4 h), and oral bioavailability (F, 10.1%) in rat models by oral administration of OPD. These data suggest that the oral bioavailability of OPD is a relatively low and thus needed to be further study for improvement of the bioavailability either structural modifications or appropriate formulation of administration of OPD.

## 3. Materials and Methods

### 3.1. Chemicals

Dulbecco’s modified Eagle’s medium (DMEM) and fetal bovine serum (FBS) were purchased from HyClone Laboratories (Logan, UT, USA). Antibiotics-antimycotics solution, Lipofectamine® RNAiMAX, negative control siRNA, and Opti-MEM® Reduced Serum Medium were purchased from Invitrogen (Grand Island, NY, USA). p53-specific siRNA was purchased from Bioneer (Daejeon, Korea). The nuclear extract kit was purchased from Active Motif (Carlsbad, CA, USA). Bovine serum albumin (BSA), dimethyl sulfoxide (DMSO), trichloroacetic acid (TCA), sulforhodamine B (SRB), propidium iodide (PI), ribonuclease A (RNase A), and other agents were purchased from Sigma-Aldrich (St. Louis, MO, CA). Antibodies against p-chk1 (Ser345) (#2348), chk1 (#2360), p-cdc25c (Ser198) (#9529), cdc25c (#4688), p-cdc2 (Tyr15) (#9111), p-cdc2 (Thr161) (#9114), p-p53 (Ser15) (#9284), p21 (#3733), and α/β tubulin (#2148) were purchased from Cell Signaling (Danvers, MA, USA). Antibodies against cyclin B1 (#752), cdc-2 (#54), β-actin, p53 (#126), MDM2 (#965), GADD45α (#797), lamin B1 (#20682) were obtained from Santa Cruz Biotechnology (Dallas, TX, USA)

OPD (Figure 1) and its analogs, isolated from the root of *Angelica dahurica*, were provided by Dr. Jin-Woong Kim (College of Pharmacy, Seoul National University, Seoul, Korea). *Angelica dahurica* (9 kg, dry weight) was purchased from Kyungdong Market Herbal Medicine in Seoul, and ultrasonically extracted three times for 120 min with 100% MeOH. The extract was filtered and concentrated under reduced pressure to obtain MeOH extract (910 g), which was suspended in distilled water to obtain a CHCl_3_ fraction (229 g). OPD and its analogs were obtained by various column chromatography. Each component was analyzed by ^1^H-NMR, ^13^C-NMR, and FABMS, and then identified compared with the literature values of corresponding compounds. OPD and its analogs, dissolved in 100% DMSO.

### 3.2. Cell Culture

SK-Hep-1, HepG2, and Hep3B cells were purchased from the American Type Culture Collection (Manassas, VA, USA). Cells were cultured in DMEM supplemented with 10% heat-inactivated fetal bovine serum (FBS) and antibiotics-antimycotics (PSF; 100 units/mL penicillin G sodium, 100 μg/mL streptomycin, and 250 ng/mL amphotericin B). Cells were incubated in a humidified atmosphere containing 5% CO_2_ at 37 °C.

### 3.3. Cell Proliferation Assay

Cell proliferation was measured by the sulforhodamine B (SRB) assay [36]. Cells were seeded in 96-well plates (3 × 10^4^ cells/mL), incubated for 24 h, and either fixed (for zero day controls) or treated with various concentrations of test compounds (total volume of 200 μL/well) for 24, 48, and 72 h. After treatment, the cells were fixed with 50% TCA solution and dried at room temperature. Fixed cells were stained in 0.4% SRB in 1% acetic acid, and unbound dye was washed with 1% acetic acid. Stained cells were dried and dissolved in 10 mM Tris (pH 10.0). The absorbance was measured at 515 nm, and cell proliferation was determined as follows: cell proliferation (%) = (average absorbance compound – average absorbance zero day) / (average absorbance control – average absorbance zero day) × 100. IC_50_ values were calculated by non-linear regression analysis using the TableCurve 2D v5.01 software (Systat Software Inc., San Jose, CA, USA).

### 3.4. Cell Cycle Analysis

SK-Hep-1 cells were plated at a density of 1 × 10^6^ cells per 100-mm culture dish and incubated for 24 h. Fresh media containing various concentrations of test sample were added to the culture dishes. Following a 24 h incubation, the cells were harvested (trypsinization and centrifugation) and fixed with 70% ethanol overnight at 4 °C. Fixed cells were washed with PBS and incubated with a staining solution containing RNase A (50 μg/mL) and propidium iodide (50 μg/mL) in PBS for 30 min at room temperature. The cellular DNA content was analyzed with a FACSCalibur flow cytometer (BD Biosciences, San Jose, CA, USA). At least 20,000 cells were used for each analysis, and the distribution of cells in each phase of the cell cycle was displayed as histograms.

### 3.5. Western Blot Analysis

SK-Hep-1 human hepatoma cells were exposed to various concentrations of OPD for the indicated times. After incubation, the cells were lysed, and the protein concentrations were determined by the bicinchoninic acid method [37]. Each protein was subjected to 6–15% SDS-PAGE. Proteins were transferred onto PVDF membranes (Millipore, Bedford, MA, USA) by electroblotting, and membranes were blocked for 1 h with blocking buffer [5% bovine serum albumin (BSA) in tris-buffered saline-0.1% Tween 20 (TBST)] at room temperature [38]. Membranes were then incubated with indicated antibodies (mouse anti-β-actin, diluted 1:10,000; other antibodies, diluted 1:500–1:1000 in 5% BSA/TBST) overnight at 4 °C and washed three times for 10 min with TBST. After washing, membranes were incubated with corresponding secondary antibodies diluted 1:2000 in TBST for 2 h at room temperature, washed three times for 10 min with TBST, and visualized with an enhanced chemiluminescence (ECL) detection kit (LabFrontier, Suwon, Korea) using an LAS-4000 Imager (Fuji Film Corp., Tokyo, Japan).

### 3.6. RNA Interference

RNA interference of p53 was performed using siRNA duplexes purchased from Bioneer (Daejeon, Korea). The coding strand for p53 siRNA was as follows: sense CACUACAACUACA UGUGUA and antisense UACACAUGUAGUUGUAGUG. For transfection, reverse transfection was conducted using Lipofectamine RNAiMAX (Invitrogen) according to the manufacturer’s recommendations. Compound treatments occurred 24 h after transfection. Cells were harvested after 24 h and examined by western blotting.

### 3.7. Combination Assay

Determination of the effect of combination therapy was performed using the SRB assay. On day 1, 3000 cells/well in a volume of 100 μL were plated in 96-well plates. On day 2, gemcitabine (100, 200, or 400 nM) and OPD (20, 30, or 40 μM) were each added in a volume of 50 μL, in all combinations. After 72 h, the cells were fixed with 50% TCA solution for 30 min at 4 °C, rinsed 5 times with water, and air-dried. Fixed cells were colored with 80 μL of 0.4% sulforhodamine B in 0.1% acetic acid) rinsed with 0.1% acetic acid, and air dried. Sulforhodamine was redissolved in 200 μL/well of 10 mM Tris, pH 10, and the absorbance was measured at 515 nm. After calculating the percent of inhibition by OPD and gemcitabine, the combination index (CI) was estimated to evaluate the synergistic effect of OPD and gemcitabine:

$$\mathrm{CI}=\frac{{\left(D\right)}_{1}}{{\left(D_{m}\right)}_{1}{\left[f_{a}/\left(1-f_{a}\right)\right]}^{\frac{1}{m_{1}}}}+\frac{{\left(D\right)}_{2}}{{\left(D_{m}\right)}_{2}{\left[f_{a}/\left(1-f_{a}\right)\right]}^{\frac{1}{m_{2}}}}$$

where D = dose; Dm = median-effect dose; m = kinetic order; fa = fraction affected.

The combination index (CI) is a quantitative measure based on the mass-action law of the degree of drug interaction in terms of synergism and antagonism (Table 3) for a given endpoint of the effect measurement.

### 3.8. Statistical Analysis

All experiments were repeated at least three times. Data are presented as the means ± standard error (SE) for the indicated number of independently performed experiments and analyzed using Student’s *t*-test. Values of *p* < 0.05 were considered statistically significant.

## 4. Conclusions

**Figure 7:**
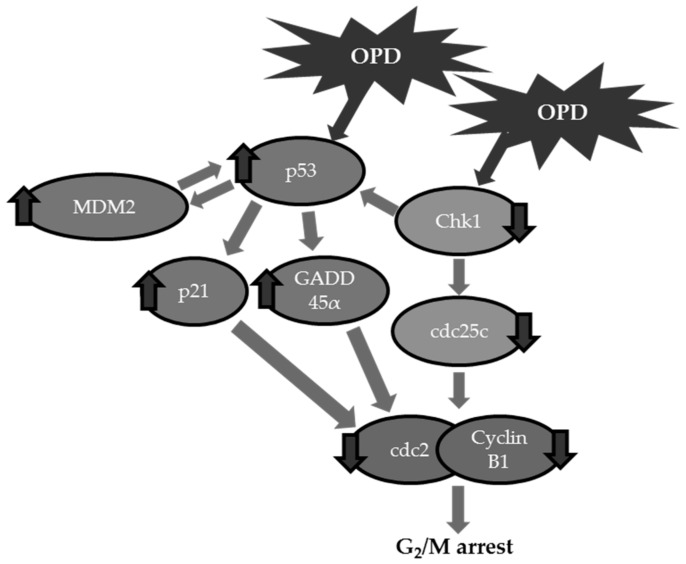
Schematic representation of the mechanisms of action of OPD against SK-Hep-1 human hepatoma cells.

In summary, the present study demonstrates the antiproliferative activities of OPD on SK-Hep-1 human hepatoma cells. The inhibition of proliferation of cancer cells was in part associated with cell cycle arrest at the G_2_/M phase and the tumor suppressor p53-mediated signaling pahway (Figure 7). These findings suggest that OPD is a promising new chemotherapeutic candidate for the management of human hepatoma cell treatment.

## Figures and Tables

**Table 1:** Anti-proliferative effects of furanocoumarins from *Angelica dahurica* on various human cancer cells.

Compounds	MDA-MB-231 ^a^	T47D	SNU638	SK-Hep-1	A549
Isoimperatorin	>100	>100	90.2	>100	>100
Byakangelicol	74.7 ^b^	46.0	50.0	72.1	41.1
Oxypeucedanin	50.8	95.5	50.4	32.4	46.3

^a^ Cancer cell line: MDA-MB-231 (breast), T47D (breast), SK-Hep-1 (liver), A549 (lung), SNU638 (stomach), ^b^ IC_50_ value: μM.

**Table 2:**
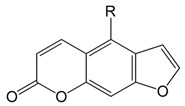
Anti-proliferative effects of oxypeucedanin analogs on SK-Hep-1 cells.

Compounds	R	IC_50_ (μM)
Oxypeucedanin	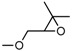	32.4
Isooxypeucedanin	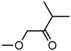	91.5
Oxypeucedanin hydrate	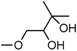	81.0
Oxypeucedanin methanolate	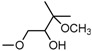	77.4

**Table 3:** Description and symbols of synergism or antagonism in drug combination studies analyzed with the combination index method.

Range of Combination Index	Description	Symbols
<0.1	Very strong synergism	+++++
0.1–0.3	Strong synergism	++++
0.3–0.7	Synergism	+++
0.7–0.85	Moderative synergism	++
0.85–0.90	Slight synergism	+
0.90–1.10	Nearly addictive	±
1.10–1.20	Slight antagonism	−
1.20–1.45	Moderate antagonism	−−
1.45–3.3	Antagonism	−−−
3.3–10	Strong antagonism	−−−−
>10	Very strong antagonism	−−−−−

## References

[1] Jemal A., Bray F., Center M.M., Ferlay J., Ward E., Forman D. (2011). Global cancer statistics. Ca. Cancer. J. Clin..

[2] Llovet J.M., Burroughs A., Bruix J. (2003). Hepatocellular carcinoma. Lancet.

[3] Bruix J., Sherman M. (2005). Management of hepatocellular carcinoma. Hepatology.

[4] Bruix J., Sherman M., Llovet J.M., Beaugrand M., Lencioni R., Burroughs A.K., Christensen E., Pagliaro L., Colombo M., Rodes J. (2001). Clinical management of hepatocellular carcinoma. Conclusions of the barcelona-2000 easl conference. European association for the study of the liver. J. Hepatol..

[5] Llovet J.M., Bruix J. (2003). Systematic review of randomized trials for unresectable hepatocellular carcinoma: Chemoembolization improves survival. Hepatology.

[6] Bai Y., Li D., Zhou T., Qin N., Li Z., Yu Z., Hua H. (2016). Coumarins from the roots of angelica dahurica with antioxidant and antiproliferative activities. J. Funct. Foods.

[7] Wall M.E., Wani M.C., Manikumar G., Hughes T.J., Taylor H., McGivney R., Warner J. (1988). Plant antimutagenic agents, 3. Coumarins. J. Nat. Prod..

[8] Cai Y., Baer-Dubowska W., Ashwood-Smith M., DiGiovanni J. (1997). Inhibitory effects of naturally occurring coumarins on the metabolic activation of benzo[a]pyrene and 7,12-dimethylbenz[a]anthracene in cultured mouse keratinocytes. Carcinogenesis.

[9] Oh H., Lee H.S., Kim T., Chai K.Y., Chung H.T., Kwon T.O., Jun J.Y., Jeong O.S., Kim Y.C., Yun Y.G. (2002). Furocoumarins from angelica dahurica with hepatoprotective activity on tacrine-induced cytotoxicity in hep g2 cells. Planta Med..

[10] Branzei D., Foiani M. (2008). Regulation of DNA repair throughout the cell cycle. Nat. Rev. Mol. Cell Biol..

[11] Goodarzi A.A., Block W.D., Lees-Miller S.P. (2003). The role of atm and atr in DNA damage-induced cell cycle control. Prog. Cell Cycle Res..

[12] Zhivotovsky B., Kroemer G. (2004). Apoptosis and genomic instability. Nat. Rev. Mol. Cell Biol..

[13] Hartwell L.H., Kastan M.B. (1994). Cell cycle control and cancer. Science.

[14] Molinari M. (2000). Cell cycle checkpoints and their inactivation in human cancer. Cell Prolif..

[15] Levine A.J. (1997). P53, the cellular gatekeeper for growth and division. Cell.

[16] Dai Y., Grant S. (2010). New insights into checkpoint kinase 1 in the DNA damage response signaling network. Clin. Cancer Res..

[17] Jessus C., Ozon R. (1995). Function and regulation of cdc25 protein phosphate through mitosis and meiosis. Prog. Cell Cycle Res..

[18] Blasina A., de Weyer I.V., Laus M.C., Luyten W.H., Parker A.E., McGowan C.H. (1999). A human homologue of the checkpoint kinase cds1 directly inhibits cdc25 phosphatase. Curr. Biol..

[19] Furnari B., Blasina A., Boddy M.N., McGowan C.H., Russell P. (1999). Cdc25 inhibited in vivo and in vitro by checkpoint kinases cds1 and chk1. Mol. Biol. Cell.

[20] Porter L.A., Donoghue D.J. (2003). Cyclin b1 and cdk1: Nuclear localization and upstream regulators. Prog. Cell Cycle Res..

[21] Chehab N.H., Malikzay A., Stavridi E.S., Halazonetis T.D. (1999). Phosphorylation of ser-20 mediates stabilization of human p53 in response to DNA damage. Proc. Natl. Acad. Sci. USA.

[22] Unger T., Juven-Gershon T., Moallem E., Berger M., Vogt Sionov R., Lozano G., Oren M., Haupt Y. (1999). Critical role for ser20 of human p53 in the negative regulation of p53 by mdm2. Embo J..

[23] Yuan J., Luo K., Zhang L., Cheville J.C., Lou Z. (2010). Usp10 regulates p53 localization and stability by deubiquitinating p53. Cell.

[24] Hall-Jackson C.A., Cross D.A., Morrice N., Smythe C. (1999). Atr is a caffeine-sensitive, DNA-activated protein kinase with a substrate specificity distinct from DNA-pk. Oncogene.

[25] Nghiem P., Park P.K., Kim Y., Vaziri C., Schreiber S.L. (2001). Atr inhibition selectively sensitizes g1 checkpoint-deficient cells to lethal premature chromatin condensation. Proc. Natl. Acad. Sci. USA.

[26] Jin L., Li C., Xu Y., Wang L., Liu J., Wang D., Hong C., Jiang Z., Ma Y., Chen Q. (2013). Epigallocatechin gallate promotes p53 accumulation and activity via the inhibition of mdm2-mediated p53 ubiquitination in human lung cancer cells. Oncol. Rep..

[27] Kang T.J., Lee S.Y., Singh R.P., Agarwal R., Yim D.S. (2009). Anti-tumor activity of oxypeucedanin from ostericum koreanum against human prostate carcinoma du145 cells. Acta Oncol..

[28] Kimura Y., Sumiyoshi M., Sakanaka M., Taniguchi M., Baba K. (2013). In vitro and in vivo antiproliferative effect of a combination of ultraviolet-a and alkoxy furocoumarins isolated from umbelliferae medicinal plants, in melanoma cells. Photochem. Photobiol..

[29] Greenblatt M.S., Bennett W.P., Hollstein M., Harris C.C. (1994). Mutations in the p53 tumor suppressor gene: Clues to cancer etiology and molecular pathogenesis. Cancer Res..

[30] Hollstein M., Sidransky D., Vogelstein B., Harris C.C. (1991). P53 mutations in human cancers. Science.

[31] Dai C., Gu W. (2010). P53 post-translational modification: Deregulated in tumorigenesis. Trends Mol. Med..

[32] Carmichael J. (1998). The role of gemcitabine in the treatment of other tumours. Br. J. Cancer.

[33] Nabhan C., Krett N., Gandhi V., Rosen S. (2001). Gemcitabine in hematologic malignancies. Curr. Opin. Oncol..

[34] Arlander S.J., Eapen A.K., Vroman B.T., McDonald R.J., Toft D.O., Karnitz L.M. (2003). Hsp90 inhibition depletes chk1 and sensitizes tumor cells to replication stress. J. Biol. Chem..

[35] Chen L., Jian Y., Wei N., Yuan M., Zhuang X., Li H. (2015). Separation and simultaneous quantification of nine furanocoumarins from radix angelicae dahuricae using liquid chromatography with tandem mass spectrometry for bioavailability determination in rats. J. Sep. Sci..

[36] Vichai V., Kirtikara K. (2006). Sulforhodamine b colorimetric assay for cytotoxicity screening. Nat. Protoc..

[37] Smith P.K., Krohn R.I., Hermanson G., Mallia A., Gartner F., Provenzano M., Fujimoto E., Goeke N., Olson B., Klenk D. (1985). Measurement of protein using bicinchoninic acid. Anal. Biochem..

[38] Byun W.S., Kim W.K., Han H.J., Chung H.-J., Jang K., Kim H.S., Kim S., Kim D., Bae E.S., Park S. (2019). Targeting histone methyltransferase dot1l by a novel psammaplin a analog inhibits growth and metastasis of triple-negative breast cancer. Mol. Ther. -Oncolytics.

